# Omics analysis of *Penaeus monodon* in response to salinity changes

**DOI:** 10.1007/s44154-024-00207-4

**Published:** 2025-02-27

**Authors:** Sheng Huang, Shigui Jiang, Song Jiang, Jianhua Huang, Qibin Yang, Lishi Yang, Jianzhi Shi, Erchao Li, Falin Zhou, Yundong Li

**Affiliations:** 1 Key Laboratory of South China Sea Fishery Resources Exploitation and Utilization, Ministry of Agriculture and Rural Affairs/South China Sea Fisheries Research Institute, Chinese Academy of Fishery Sciences, Guangzhou, 510300 China; 2 Key Laboratory of Efficient Utilization and Processing of Marine Fishery Resources of Hainan Province, Sanya Tropical Fisheries Research Institute, Sanya, 572018 China; 3 School of Life Sciences, East China Normal University, Shanghai, 200241 China; 4 Shenzhen Base of South China Sea Fisheries Research Institute, Chinese Academy of Fishery Sciences, Shenzhen, 518108 China

**Keywords:** *Penaeus monodon*, Salinity stress, Microbiome, Transcriptome, Metabolome, Shrimp

## Abstract

Salinity profoundly influences the physiological and biochemical well-being of shrimp in aquaculture, dictating their growth, survival, and overall health. In this study, research exposed *Penaeus monodon* to three different salinity environments (salinity 5, 20, and 30) for 62 days, which affected shrimp growth and the development of the gut and hepatopancreas. Further omics analyses revealed that high salinity levels led to the predominance of *Proteobacteria* and *Cyanobacteria* in the gut microbiome, enhancing the osmoregulatory and immune functions essential for shrimp recovery under salinity stress. The transcriptome highlighted the upregulation of genes involved in lipid metabolism and amino acid processing, emphasizing the critical role of lipid dynamics in salinity adaptation. Metabolomics revealed alterations in membrane lipid composition, osmoprotectant synthesis, and antioxidative defense mechanisms, which are crucial for maintaining cellular integrity under salinity stress. Integrated analysis revealed a comprehensive molecular framework for the resilience of *P. monodon* to salinity fluctuations, underscoring lipid metabolism as the core of its adaptation strategy. This study enriches understanding of salinity regulation in *P. monodon*, providing practical insights for enhancing low-salinity aquaculture and responding to environmental challenges.

**Graphical Abstract:**

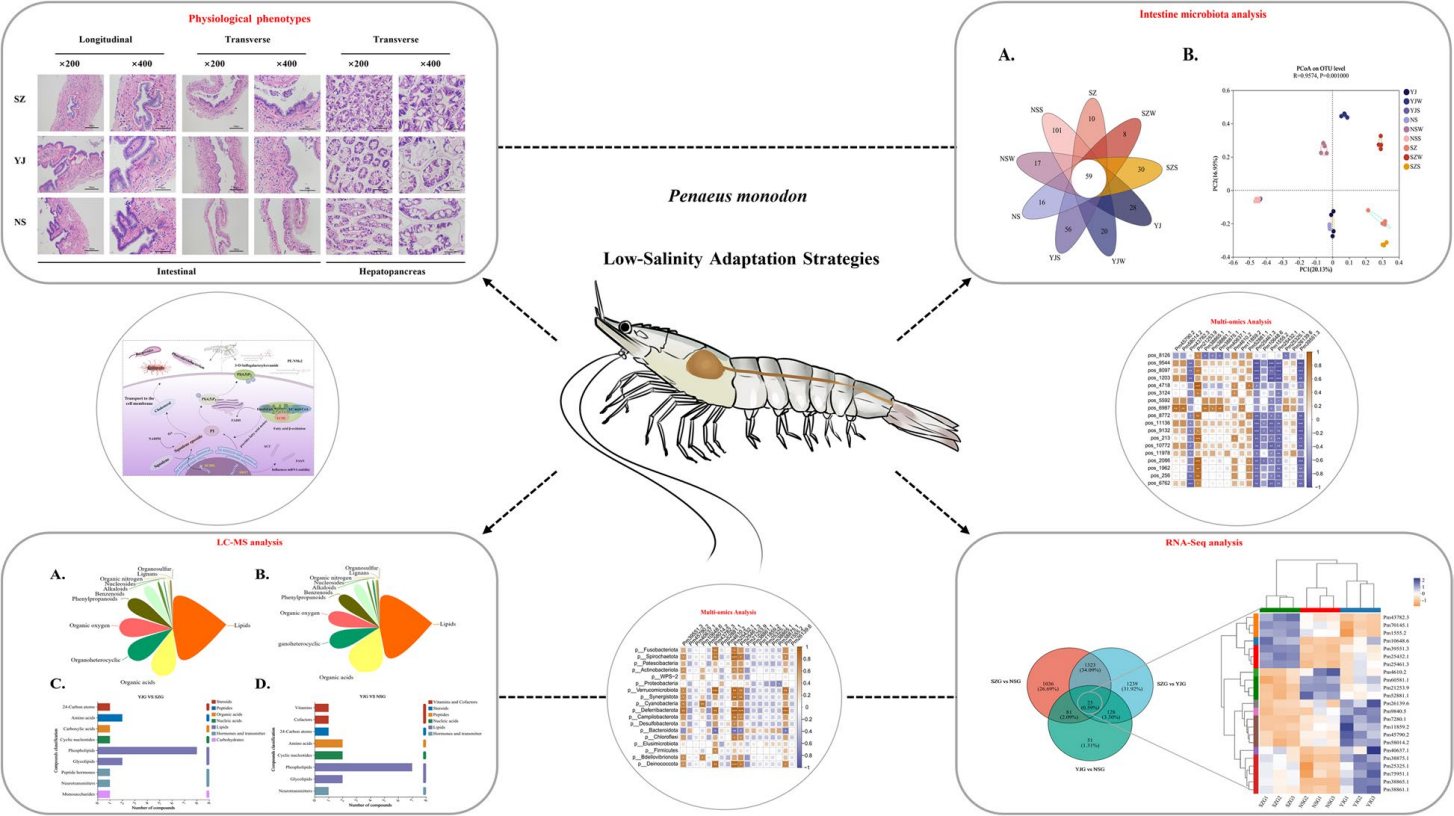

**Supplementary Information:**

The online version contains supplementary material available at 10.1007/s44154-024-00207-4.

## Introduction

*Penaeus monodon*, commonly known as the black tiger shrimp, holds a significant place in global aquaculture due to its exceptional euryhalinity and ability to thrive across a wide range of salinities (Kaeodee et al. 2011). This remarkable adaptability enables the species to inhabit and reproduce in vast geographic areas, from the Sea of Japan to the west coast of Africa. The unique ability of black tiger shrimp to grow in environments with salinities ranging from 3 to 45 ppt, with an optimal growth range between 20 and 35 ppt, is rooted in its complex osmoregulatory mechanisms (Shekhar et al. 2013). These mechanisms allow for survival under varying salinity conditions, although both the growth rate and disease resistance decline under extreme salinity levels, high or low, potentially due to inadequate energy intake, although the specific mechanisms involved remain to be fully understood (Si et al. 2022).

Recent research has highlighted the significant impact of diet, nutritional status, and developmental stage on the gut microbial communities of aquatic animals (Wei et al. 2018). While early studies indicated the role of microbiota, such as those in *E. sinensis*, *P. trituberculatus*, and *Lateolabrax japonicus*, in promoting the salinity tolerance of shrimp, focusing mainly on the influence of biotic factors on the gut microbiome of aquatic animals, these studies often overlooked abiotic factors such as salinity. Research on how nonbiological factors affect the composition of aquatic animal gut microbiota is still in its nascent stages.

Under salinity stress, shrimp deploy a sophisticated adaptation strategy through a combination of physiological and molecular mechanisms. This process involves crucial cellular components such as the Na^+^/K^+^-ATPase and ion channels for Ca^2+^, K^+^, and Cl^-^, which maintain electrolyte balance and osmotic pressure, ensuring stability across varying salinities (Yuan et al. 2021). Key signaling pathways, such as the MAPK, PI3K/Akt, and NF-κB pathways, are activated to regulate gene expression, affecting everything from stress responses to cell survival and proliferation (Shekhar et al. 2013). These pathways help in managing external salinity variations, promoting adaptability, and enhancing survival rates (Li et al. 2019). Furthermore, activation of the Nrf2-ARE pathway bolsters antioxidant defenses, mitigating oxidative stress and improving cellular antioxidant capacity (Chen et al., 2015).

In the realm of shrimp aquaculture, addressing the challenges posed by low salinity is crucial. Research indicates that shrimp raised under prolonged exposure to low salinity without adequate dietary nutrition adjustments are more prone to environmental stresses (Li et al. 2015). This leads to inhibited growth and diminished survival rates (Chen et al., 2021b). Additionally, under conditions of salinity stress, the ability of shrimp to regulate osmotic pressure and maintain a balance of salinity and water is significantly compromised. Shrimp subjected to low-salinity conditions may also exhibit heightened vulnerability to pathogens, increasing the likelihood of infections, including white spot syndrome virus (WSSV) (Alloul et al., 2021; Van Thuong et al. 2016). Furthermore, dietary supplementation with selenium has been found to mitigate the adverse effects of low salinity on shrimp, improving their growth, antioxidative abilities, and hepatopancreatic transcriptome responses (Yu et al. 2023).

In the complex interplay between salinity conditions and shrimp physiology, the lipid profile of the Pacific white shrimp emerges as a critical factor in mediating their adaptability to fluctuating environmental salinity. Central to this adaptation are key lipid constituents, phosphatidylcholine (PC), phosphatidylinositol (PI), phosphatidic acid (PA), phosphatidylethanolamine (PE), and triglycerides (TG), which remain prevalent within the gills and muscles across varying salinity levels (Huang et al., 2019). These lipids play an indispensable role in maintaining cellular integrity, ensuring optimal growth, and supporting lipid metabolism and health status under salinity stress (Chen et al., 2015). Notably, dietary lipid levels are pivotal in enhancing the antioxidative and immune responses of post-larval shrimp, underscoring the necessity of tailored dietary interventions to bolster resilience against low salinity challenges (Martinez-Antonio et al., 2019). The regulation of dietary lipid intake, including the essential incorporation of phospholipids and cholesterol, has been demonstrated to significantly ameliorate stress responses and improve production performance under conditions of salinity stress. This suggests a nuanced relationship between dietary lipid composition and the shrimp ability to maintain osmotic balance and mitigate reactive oxygen species (ROS)-induced tissue damage when confronted with low salinity environments (Chuphal et al.,^ 2021^).

Expanding on established research into the euryhalinity of *Penaeus monodon* this study broadens its investigative lens to encompass not only established physiological and osmoregulatory mechanisms but also enzyme activities, histological changes, and microbial community dynamics under varying salinity stresses. Integrating metabolic, transcriptomic, and microbiomic data, the study elucidates the complex strategies this species employs to navigate salinity gradients. Detailed analysis of the interplay between diet, immune response, and gut microbiota reveals their collective impact on shrimp resilience and health. Furthermore, assessments of biochemical profiles, including activities of superoxide dismutase and catalase, alongside histological examinations of tissue responses, enrich our understanding of the shrimp's adaptations to environmental challenges. By dissecting these multilayered responses, the research highlights critical pathways and mechanisms that could guide optimized aquaculture practices, aiming to enhance the sustainability and productivity of shrimp farming in fluctuating salinity conditions. This comprehensive approach not only clarifies the immediate responses of shrimp to salinity stress but also underscores the broader adaptive capacities that facilitate their survival and growth in diverse aquatic environments.

## Results

### Measuring growth and health indicators under varying salinity conditions

#### Growth indicators

The growth parameters of *P. monodon* were evaluated at three salinity levels, revealing significant differences in response to environmental salinity. In the high-salinity environment (SZ), the weight gain of the shrimp was significantly greater than that in the low-salinity group (NS). The weight gain rates in the high-salinity and YJ groups were similar and significantly exceeded that of the low-salinity group. Moreover, the specific growth rate (SGR) in the high-salinity group was notably greater than that in the low-salinity group (Table 1).

**Table 1:** Measurement of Growth Indicators in *P. Monodon*

No	Group	Weight gain(g)	Weight gain rate(%)	Specific growth rate (%/d)
1	SZ	15.91 ± 0.95^a^	1302.25 ± 93.02^a^	4.21 ± 0.10^a^
2	YJ	15.09 ± 0.78^a^	1265.81 ± 109.49^a^	4.15 ± 0.12^a^
3	NS	7.93 ± 0.42^b^	673.51 ± 60.78^b^	3.23 ± 0.12^b^

Table 1 presents the growth metrics of *Penaeus monodon* across different salinity treatments. Data are presented as mean ± standard deviation (SD) based on ten biological replicates per treatment. Different lowercase letters (a, b) denote significant differences between salinity treatments (*P* < 0.05)

#### Health indicators

**Fig. 1:**
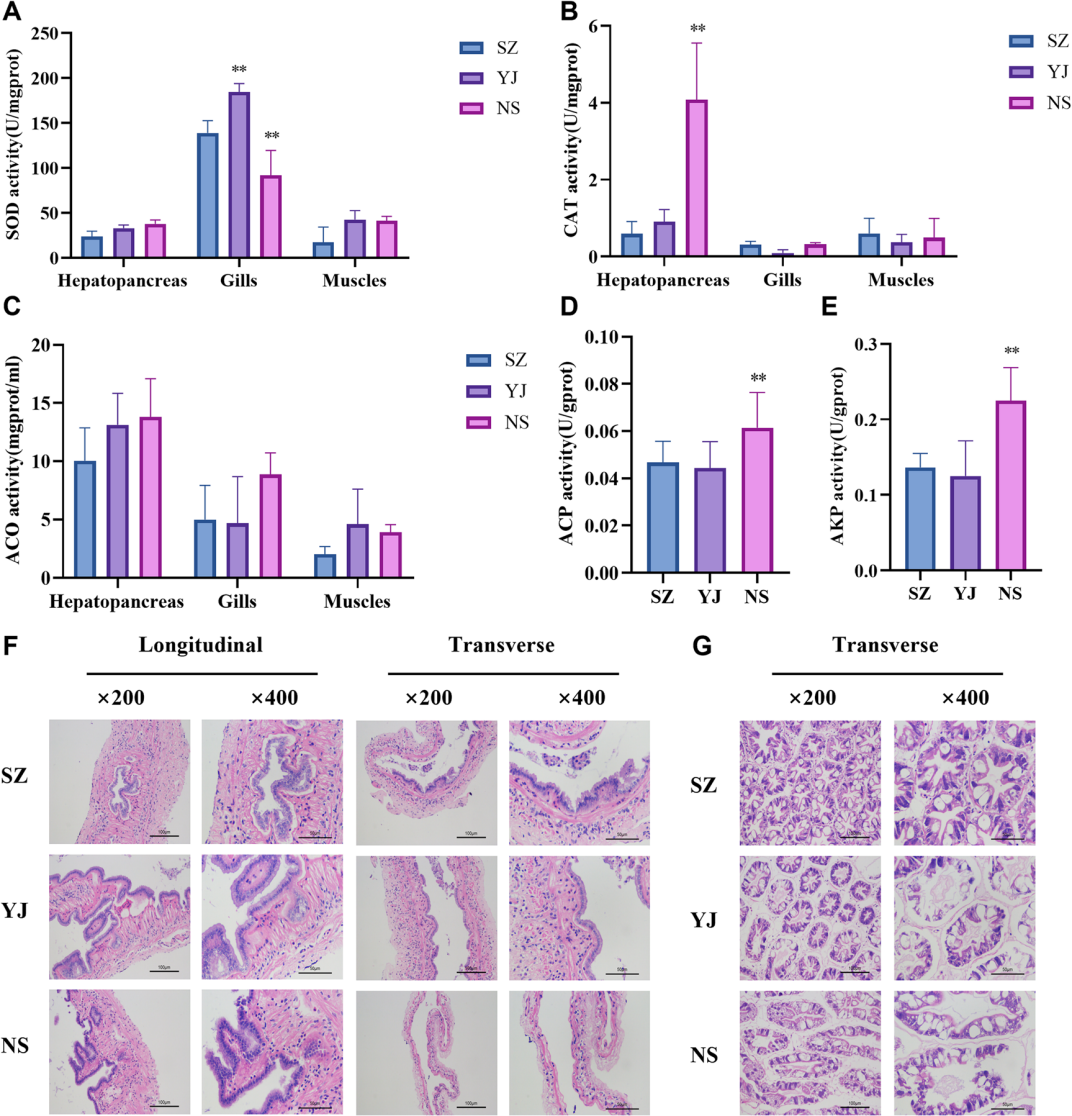
Biochemical markers and phenotypic identification of *P. monodon* under salinity stress: **A** activity of superoxide dismutase (SOD) enzyme, **B** catalase (CAT) enzyme activity, **C** 1-aminocyclopropane-1-carboxylate oxidase (ACO) enzyme activity, **D** acid phosphatase (ACP) enzyme activity, **E** alkaline phosphatase (AKP) enzyme activity, **F** cross and longitudinal sections of intestinal tissues in paraffin embedding, cross sections of hepatopancreatic tissues in paraffin embedding. The p value is calculated by student t test and two-tailed. *, *P* < 0.05; **, *P* < 0.01

Tissue samples from the hepatopancreas, gills, and intestines of shrimp grown under varying salinity conditions were analyzed to determine their oxidative and metabolic levels. Significant changes were observed in the superoxide dismutase (SOD) enzyme levels within the gills across medium (YJ) and low (NS) salinity conditions. SOD enzyme levels in the hepatopancreatic tissue reached 4.08 U/mg prot in the low-salinity group, markedly exceeding the 0.59 U/mg prot observed in the low-salinity group. The enzymatic activities of acid phosphatase (ACP) and alkaline phosphatase (AKP) also increased significantly, with low-salinity group levels of 0.06 U/gprot and 0.23 U/gprot, respectively, surpassing those in the YJ group (0.04 U/gprot and 0.13 U/gprot) (Fig. 1A-E).

The intestinal tissue morphology of *P. monodon* was analyzed, revealing the formation of inward-projecting folds and numerous microvilli on the surface of these folds, which contained a high number of cell nuclei. In the low-salinity group, the connective tissue layers were more loosely organized around muscle bundles, with a thinner muscle layer enclosed by an outer membrane layer. Compared to those in the high-salinity group, an increase in salinity concentration led to a marked increase in the number and dimensions of intestinal folds, reducing the intestinal lumen space and resulting in more tightly packed cells within the lumen, as well as an abundance of connective tissue with greater density than that observed in the low-salinity group. Morphological analysis of the hepatopancreatic tissue in the high-salinity group revealed a normal cellular architecture with tightly connected cells and centrally located stellate lumens. As salinity decreased, the YJ group displayed irregular contraction of stellate structures, and in the low-salinity group, lumen structures became enlarged, with stellate structures nearly disappearing, vacuolation increasing, and tissue edges becoming thinner (Fig. 1F).

### Intestinal microbiota

#### Richness and diversity

**Fig. 2:**
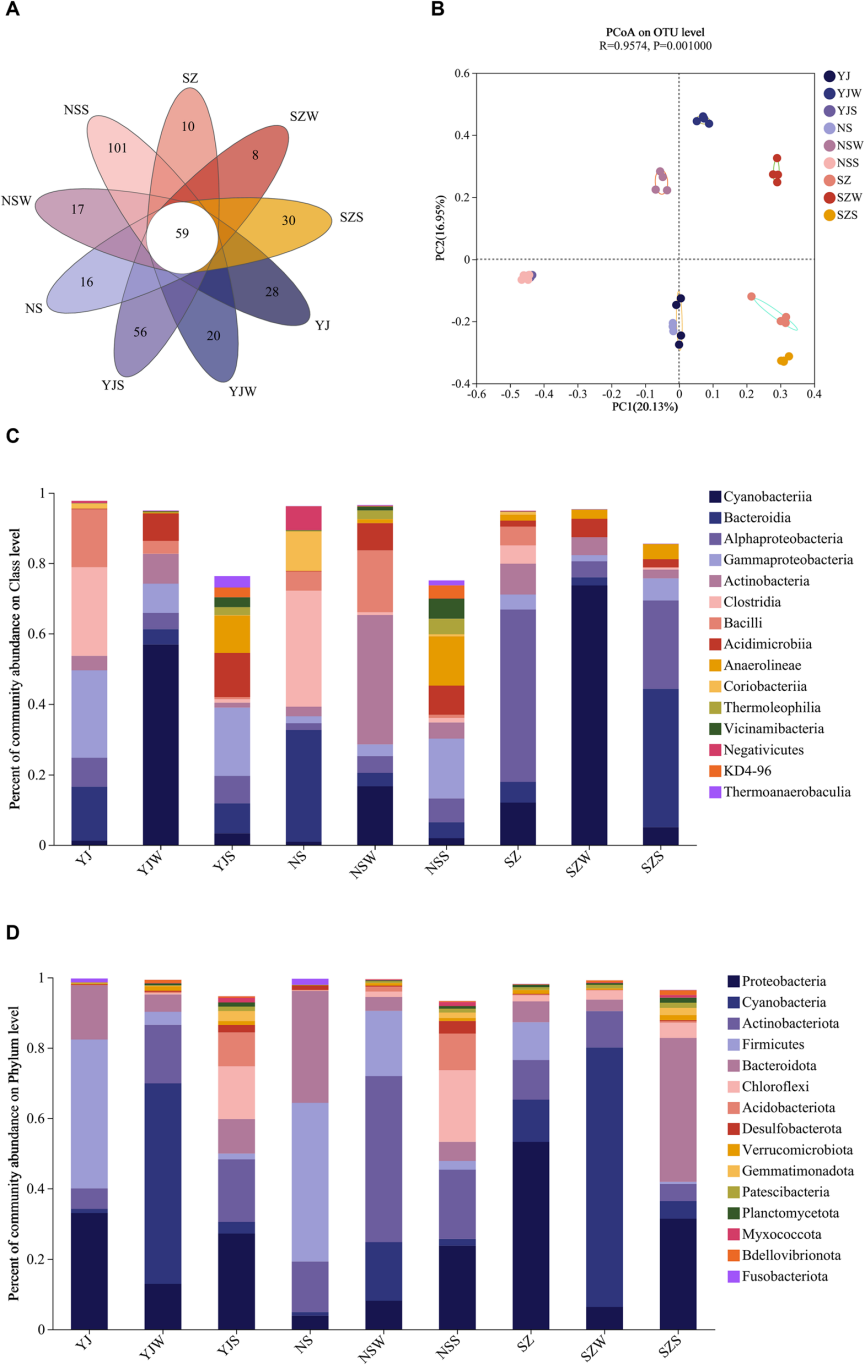
Microbial Community and Habitat Composition in the Intestine of *P. monodon* under Salinity Stress: **A** Venn diagram showing the overlap between intestinal and habitat microbial communities; **B** Principal Coordinates Analysis (PCoA) of Operational Taxonomic Units (OTUs) across different salinity concentrations in *P. monodon*; **C**-**D** Average relative abundance of dominant bacterial phyla and genera in *P. monodon* across different salinity concentrations

**Fig. 3:**
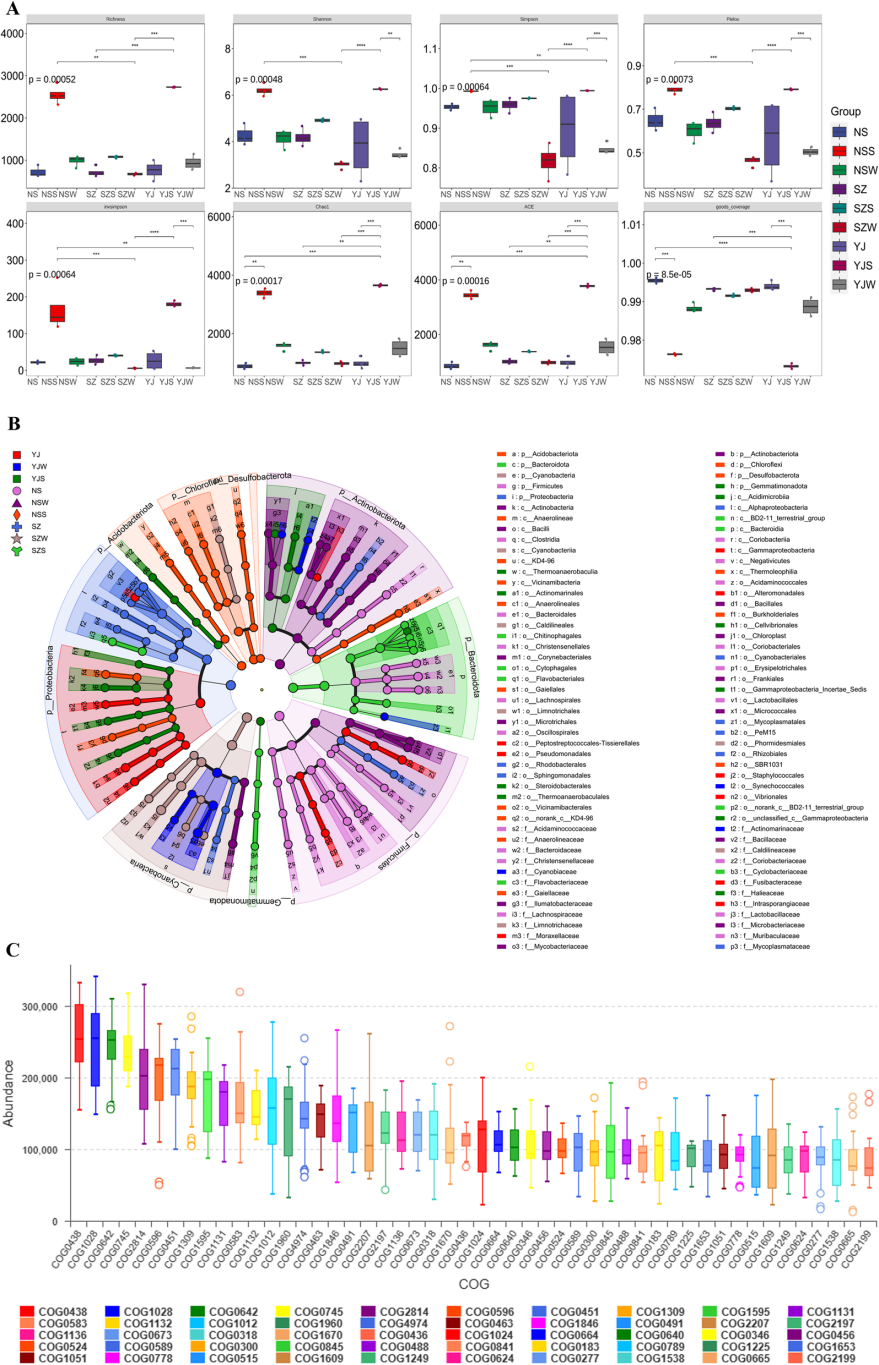
Composition and COG Analysis of Microbial Communities in the Intestine, Soil, and Seawater of *P. monodon* Under Salinity Stress: **A** Alpha Diversity of Microbial Communities in the Intestine and Habitat of *P. monodon* Across Different Salinity Concentrations. **B** LEfSe (linear discriminant analysis effect size) cladogram illustrating the phylogenetic distribution of microbiota associated with different environments. **C** Abundance statistics of COG (clusters of orthologous groups) functions, reflecting the metabolic capabilities of the microbial communities under salinity stress conditions

Microbiome analysis yielded a total of 1,827,838 reads across all microbial samples. Rarefaction curve analysis revealed that, compared to the control group (YJ), both the low-salinity group (NS) and the high-salinity group (SZ) exhibited a decrease in the Chao1 index, with NS having a higher Chao1 index than SZ. Similarly, the Simpson index was significantly greater in YJ than in both NS and SZ. The Shannon index did not significantly change (Fig. 3A). There were also significant differences in the number of unique OTUs among YJ, NS, and SZ (Fig. 2A). Principal coordinate analysis (PCoA) illustrated a clear separation in community composition among samples from different groups in water, sediment, and intestinal samples (Fig. 2B).

#### Microbial composition in the water, sediment, and intestine

Analysis of the microbial composition in the water, sediment, and intestine of the three groups of shrimp revealed similar dominant microbial groups with varying abundances. At the phylum level, the relative abundances of Proteobacteria and Cyanobacteria increased with increasing salinity, whereas *those of Actinobacteria* and *Firmicutes* decreased (Fig. 2C). At the species level within the shrimp intestine, *Ruegeria*, *Parasphingopyxis*, and *Nautella* were enriched in SZ; *Ferrimonas* was enriched exclusively in YJ; and *Paenisporosarcina*, *Anaerolineaceae*, and *Steroidobacteraceae* were enriched only in NS. At the class level, the relative abundances of *Cyanobacteriia* and *Alphaproteobacteria* increased with increasing salinity, Actinobacteria increased in the intestine and water but decreased in the sediment with increasing salinity, and Clostridia decreased with increasing salinity, whereas *Gammaproteobacteria* reached its peak at moderate salinity (Fig. 2D).

#### Changes in intestinal bacterial species

LEfSe abundance difference analysis of the intestinal microbiota of YJ, NS, and SZ patients revealed that the phylum Proteobacteria contributed most significantly to SZ. At the order level, *Thalassobaculales* and *Parasphingopyxis* made prominent contributions to SZ, while the phylum *Actinobacteriota* was significant for the low-salinity group NS, with *Conexibacter* standing among the *Actinobacteriota* (Fig. 3B). Predictive functional analysis of the intestinal microbiota using PICRUSt, based on COG classification, revealed that RNA processing and modification increased with salinity, whereas carbohydrate transport and metabolism and translation, ribosomal structure, and biogenesis decreased with increasing salinity in the intestine, water, and sediment (Fig. 3C).

### Intestinal transcriptome analysis

#### Identification and functional annotation of DEGs

**Fig. 4:**
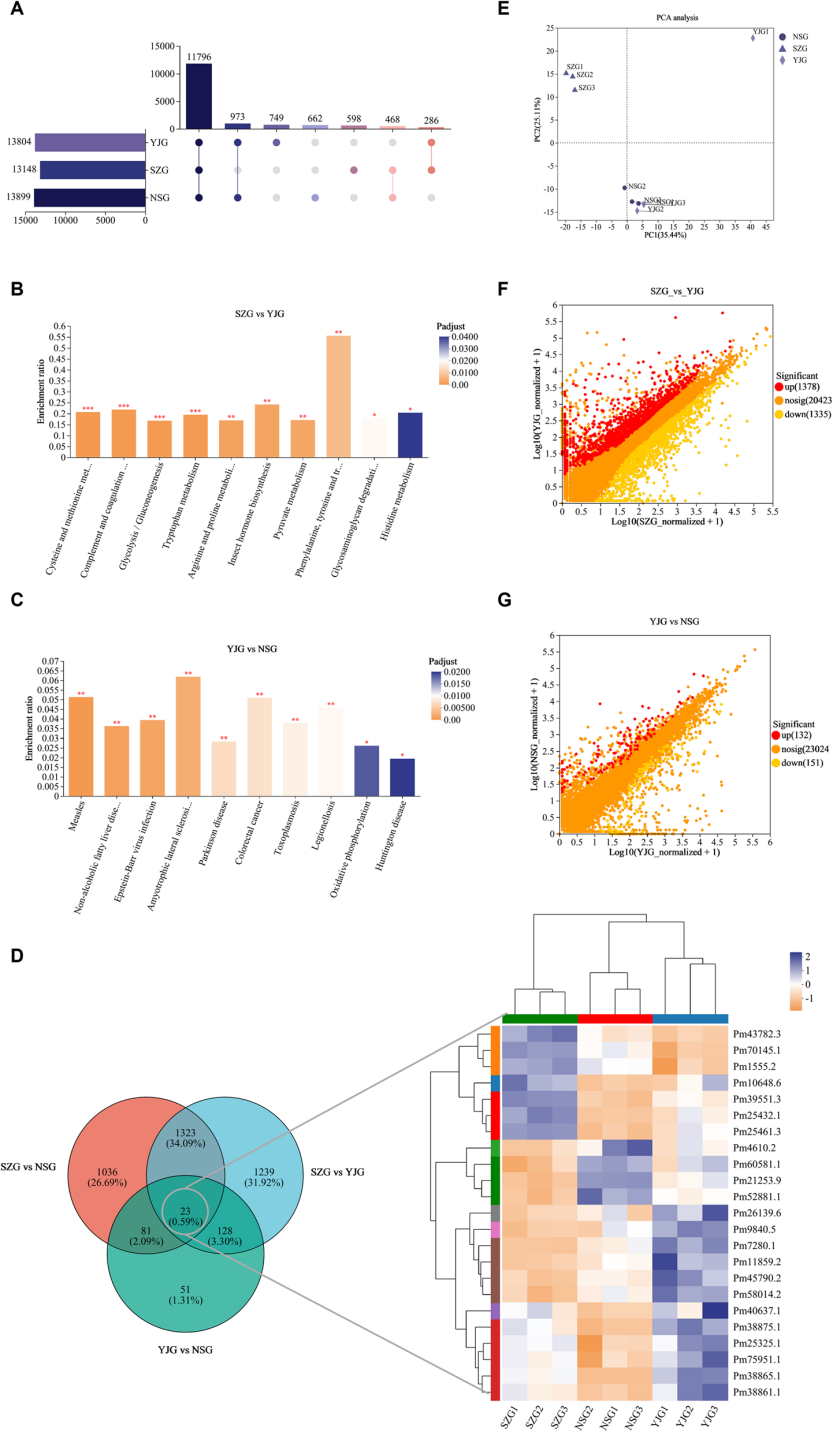
Scatter plots and Venn diagrams of differentially expressed genes (DEGs) in the intestine of *P. monodon* following salinity stress: **A** UpSet plot illustrating the intersection of DEGs across samples at different salinity concentrations; **B**-**C** stratified enrichment analysis of DEGs in Kyoto Encyclopedia of Genes and Genomes (KEGG) metabolic pathways; **D** heatmap analysis of the common DEGs in the hepatopancreas of *P. monodon* under various salinity conditions; **E** principal component analysis (PCA) of samples, assessing the variance in gene expression due to salinity stress; **F**-**G** scatter plots showing the differential expression of genes, highlighting upregulated and downregulated genes under varying salinity conditions

RNA-Seq analysis of the tiger shrimp intestine generated 72.52 Gb of clean data, with each sample reaching over 7.78 Gb of clean data and a Q30 base percentage above 95.64%. A total of 46,172 expressed genes and 86,611 transcripts were detected. Compared to those in the YJ group, 2,996 DEGs were identified in the NS and SZ hepatopancreases (Fig. 4A), with 13 DEGs present in all groups and 23 DEGs, including the putative serine/threonine-protein kinase SBK1, chloride channel protein, putative transketolase, anti-lipopolysaccharide factor, and crustacyanin (Table 2). SZ had 1,378 upregulated genes and 1,335 downregulated genes, while NSG had 132 upregulated genes and 151 downregulated genes (Fig. 4F, G).

**Table 2:** Transcriptome profile of common differentially expressed genes (DEGs) in the hepatopancreas of *P. monodon* under various salinity concentrations

No	Gene Name	Gene Description
1	Pm39551.3	putative serine/threonine-protein kinase SBK1
2	Pm21253.9	chloride channel protein 2
3	Pm25432.1	anionic trypsin-like
4	Pm60581.1	myophilin-like
5	Pm52881.1	unknown
6	Pm10648.6	unknown
7	Pm43782.3	uncharacterized protein LOC113815546
8	Pm70145.1	putative transketolase-like protein 2 isoform X1
9	Pm25461.3	putative transmembrane protease serine 9-like
10	Pm1555.2	aldehyde dehydrogenase, dimeric NADP-preferring-like
11	Pm4610.2	uncharacterized protein LOC113811243
12	Pm25325.1	anti-lipopolysaccharide factor isoform 2
13	Pm40637.1	Anti-lipopolysaccharide factor isoform 1
14	Pm45790.2	dual oxidase maturation factor 1-like
15	Pm75951.1	uncharacterized protein LOC113823074
16	Pm38865.1	crustacyanin A, partial
17	Pm38875.1	crustacyanin C3, partial
18	Pm58014.2	hypothetical protein C7M84_000730
19	Pm11859.2	open rectifier potassium channel protein 1-like isoform X1
20	Pm7280.1	dynein light chain 1
21	Pm38861.1	crustacyanin subunit A
22	Pm9840.5	adenosine deaminase 2-like
23	Pm26139.6	putative peptidyl-prolyl cis–trans isomerase 5

KEGG pathway analysis further analyzed all DEGs. Compared to those in YJG, the enriched pathways in SZ included cysteine and methionine metabolism, complement and coagulation cascades, glycolysis/gluconeogenesis, and tryptophan metabolism. In the NSG, the enriched pathways were amyotrophic lateral sclerosis (ALS), measles, nonalcoholic fatty liver disease (NAFLD), Epstein‒Barr virus infection, Parkinson disease, toxoplasmosis, and legionellosis. Compared to those in the NSG group, the pathways enriched in the SZ group were involved in lysosomes, prion diseases, and antigen processing and presentation (Fig. 4B, C).

Lipid-related DEGs showed significant regulatory shifts in response to salinity stress. These genes play a pivotal role in modulating the intricate balance of lipid metabolism, underscoring the adaptative mechanisms of *P. monodon* to fluctuating salinity levels. The analysis revealed a complex interplay between lipid metabolism and the shrimp ability to maintain cellular homeostasis and energy efficiency under stress conditions, highlighting the critical role of lipid-related pathways in the shrimp resilience and adaptability to environmental changes (Table 3).

**Table 3:** Transcriptome profile of lipid-related Differentially Expressed Genes (DEGs) in the Hepatopancreas of *P. monodon* under different salinity concentrations

Gene id	Gene description	SZvsYJ	NSvsYJ
Pm58102.1	acyl-CoA dehydrogenase 6	down	down
Pm41560.2	ATP-dependent RNA helicase DDX27	up	up
Pm34499.1	SC5DL	up	up
Pm14756.3	diacylglycerol O-acyltransferase 1-like	up	up
Pm8481.1	ECH1	up	up
Pm10870.1	Peroxisomal 3,2-trans-enoyl-CoA isomerase	up	up
Pm25850.3	venom carboxylesterase-6-like	up	up
Pm47506.1	cholinesterase 1-like isoform X2	up	up
Pm65620.1	prostaglandin E synthase 2	up	down
Pm77226.1	Phosphatidylserine synthase 2	down	down
Pm35819.2	glycine receptor subunit alpha-4-like isoform X1	down	down
Pm60716.1	methylmalonate-semialdehyde dehydrogenase	down	down
Pm26931.8	isovaleryl-CoA dehydrogenase, mitochondrial	up	up
Pm55446.7	hypothetical protein C7M84_018746	up	up
Pm28182.1	Gamma-interferon-inducible lysosomal thiol reductase	up	up

#### Identification of lipid-related DEGs

Based on annotations from the NR database, this research identified several DEGs related to lipid metabolism, providing a foundation for a better understanding of lipid metabolism in *P. monodon* under salinity stress. Fifteen lipid metabolism-related DEGs were shared between the NS and SZ patients. Compared to those in YJ, there were ten commonly upregulated DEGs, including ATP-dependent RNA helicase DDX27, SC5DL, diacylglycerol O-acyltransferase 1-like, ECH1, peroxisome 3,2-trans-enoyl-CoA isomerase, venom carboxylesterase-6-like, cholinesterase 1-like isoform X2, isovaleryl-CoA dehydrogenase, mitochondrial, hypothetical protein C7M84_018746, and gamma-interferon-inducible lysosomal thiol reductase. The expression of four DEGs, namely, acyl-CoA dehydrogenase 6, phosphatidylserine synthase 2, glycine receptor subunit alpha-4-like isoform X1, and methylmalonate-semialdehyde dehydrogenase, was downregulated. One DEG, prostaglandin E synthase 2, was upregulated in SZ patients and downregulated in NS patients (Table 3).

### Hemolymph metabolome analysis

#### Multivariate analysis of metabolite profiles

Metabolomic analysis was used to explore the changes in the hepatopancreas metabolome of *P. monodon* at different salinity concentrations. LC‒MS analysis revealed 655 metabolites in positive ion mode and 636 in negative ion mode. PLS-DA score plots and permutation tests further showed significant differences between the three groups in both positive and negative ion modes, indicating that different salinity environments induced changes in the metabolic phenotype of *the hepatopancreas of P. monodon*. MS/MS analysis revealed a total of 1,075 metabolites in shrimp hemolymph, with lipids and lipid-like molecules accounting for 38.05%, followed by organic acids and derivatives (27.35%) and organoheterocyclic compounds (10.70%) (Fig. 7A).

#### Identification and functional annotation of Differentially Metabolized (DM) genes

**Fig. 5:**
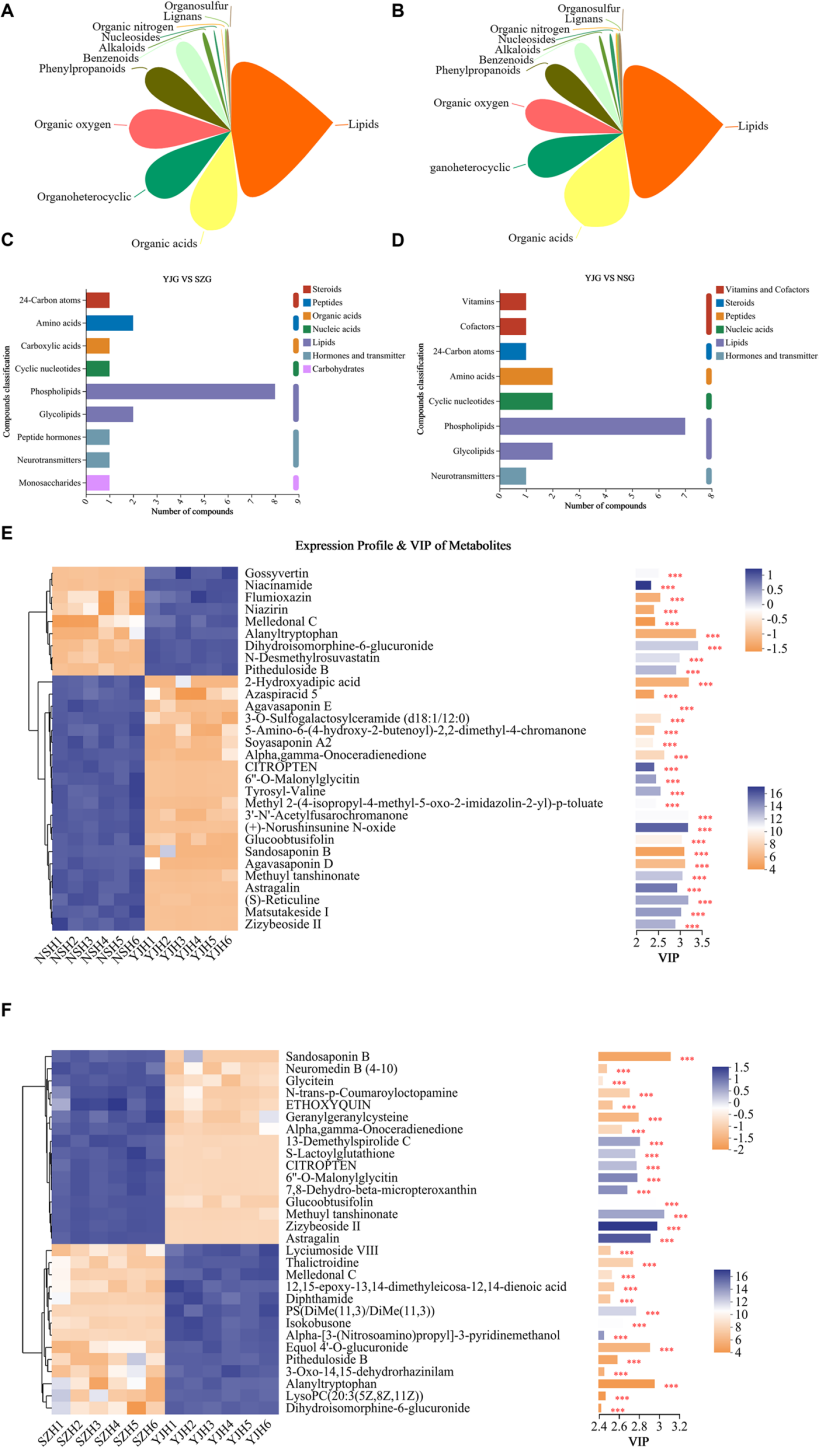
Differential metabolic analysis and investigation of variable importance in projection (VIP) values for differentially abundant metabolites in the hepatopancreas of *P. monodon* across various salinity habits: **A**-**B** Proportional analysis of differentially abundant metabolites between two samples; **D**-**F** KEGG compound classification; **E**–**F** Analysis of variable importance in projection (VIP) values

Further screening identified DMs between YJ and SZ or NS. Compared to YJ, SZ had 110 upregulated DMs and 147 downregulated DMs, and NSG had 94 upregulated DMs and 100 downregulated DMs (Fig. 7B, C). To explore the potential metabolic pathways for high and low salinity tolerance in *P. monodon*, all DMs were further analyzed through KEGG annotation. Compared to those in YJ, the most enriched pathways in SZ were bile secretion, D-arginine and D-ornithine metabolism, choline metabolism and arginine and proline metabolism; for NS, they were bile secretion, D-arginine and D-ornithine metabolism, choline metabolism and glutathione metabolism. Compared between SZ and NS, the main enriched pathways were bile secretion and choline metabolism (Fig. 5A, B).

In both the high-salinity SZ group and the low-salinity NSG, the levels of certain DMs related to membrane lipid metabolism, osmoregulatory protection, and antioxidation, including 3-O-sulfogalactosylceramide (d18:1/20:0), PE-NMe2 (15:0/22:2(13Z,16Z)), CDP-DG (18:1(11Z)/20:4(5Z,8Z,11Z,14Z)), soyasaponin V, and notoginsenoside J, increased. However, PE-NMe2(20:0/22:6(4Z,7Z,10Z,13Z,16Z,19Z)) increased in SZ but decreased in NS. PE(18:1(9Z)/0:0), PE(P-18:0/22:6(4Z,7Z,10Z,13Z,16Z,19Z)), Humilixanthin, and Tocopheronic acid were only increased in SZ, while PE-NMe2(9D3/9D3) was exclusively increased in NS (Table 4).

**Table 4:** Changes in Lipid Metabolism in the Hepatopancreas of *P. monodon* Under Various Salinity Concentrations

No	Metabolite name	Log2-fold change		Categories
		SZGvsYJG	NSGvsYJG	
1	3-O-Sulfogalactosylceramide (d18:1/20:0)	1.45	1.3599	Lipid
2	PE-NMe2(20:0/22:6(4Z,7Z,10Z,13Z,16Z,19Z))	1.1636	0.8777	Lipid
3	CDP-DG(18:1(11Z)/20:4(5Z,8Z,11Z,14Z))	1.2517	1.2604	Lipid
4	PE(18:1(9Z)/0:0)	1.0819	**/**	Lipid
5	PE(P-18:0/22:6(4Z,7Z,10Z,13Z,16Z,19Z))	1.091	**/**	Lipid
6	(E)−11-Hexadecenoic acid	1.2291	1.1841	Lipid
7	(Z)−13-Octadecenoic acid	1.0935	**/**	Lipid
8	Pregnanetriol	1.2154	1.1348	Lipid
9	Crocin 3	1.1725	1.1422	Lipid
10	Humilixanthin	1.4375	**/**	Organic acid
11	Soyasaponin V	1.4566	1.5028	Organic acid
12	Notoginsenoside J	1.2895	1.3547	Organic acid
13	Trigofoenoside G	1.1554	1.2428	Lipid
14	Tocopheronic acid	1.0933	**/**	Organic acid
15	PE-NMe2(9D3/9D3)	**/**	1.3848	Lipid
16	PE-NMe2(15:0/22:2(13Z,16Z))	1.1764	1.102	Lipid
17	4-hydroxyoct-4-enoylglycine	1.1081	1.1255	Organic acid
18	Fosinoprilat	1.1122	1.1234	**/**

### Relationships between the gut microbiota and lipid metabolism and DEGs

**Fig. 6:**
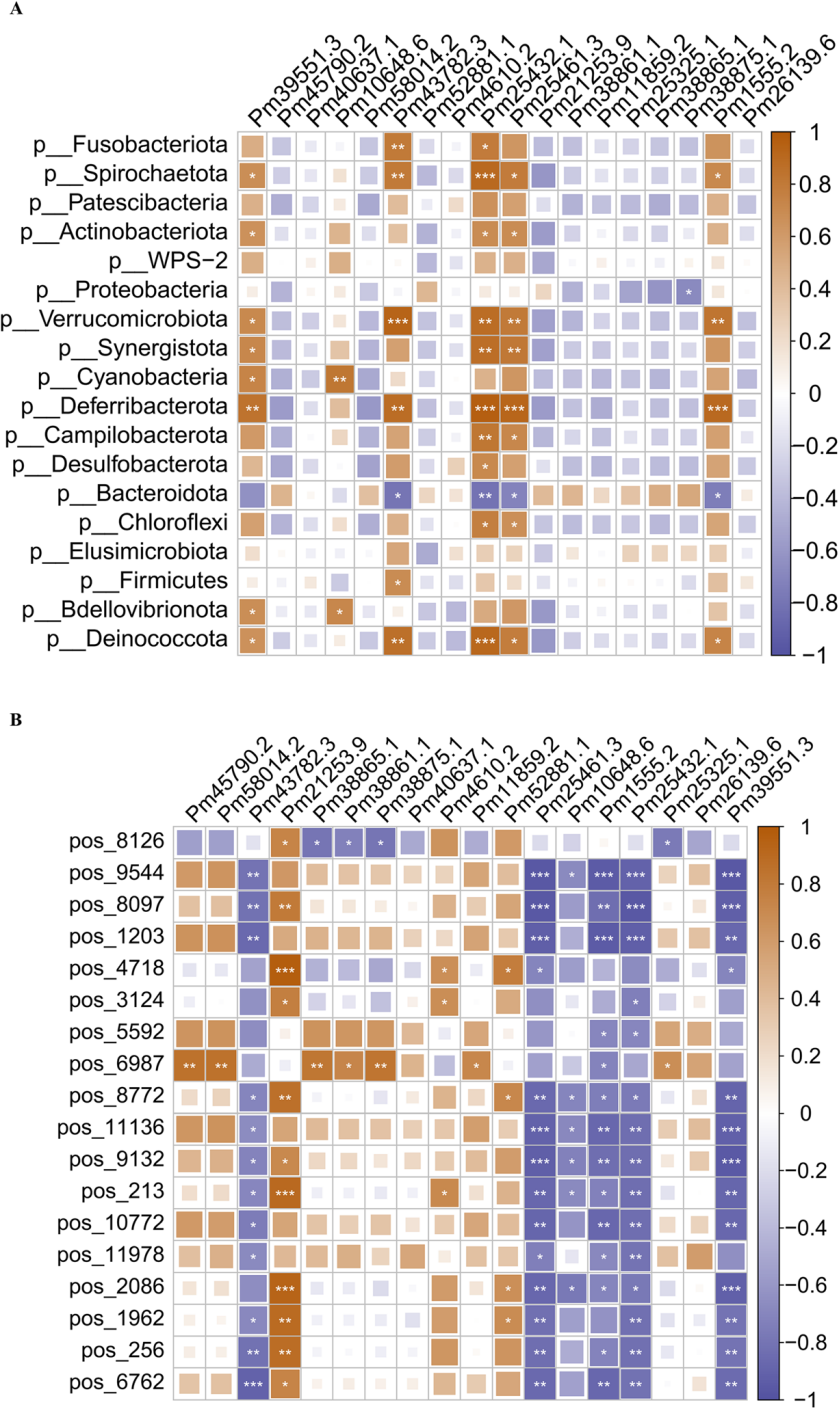
Gut bacteria exhibit significant correlations with differentially expressed genes (DEGs) and dysregulated metabolites (DMs). **A** Correlations between lipid metabolites and DEGs. **B** Correlations between gut bacteria and DEGs. Correlation coefficients are denoted by different colors (red for positive correlation; blue for negative correlation). Asterisks indicate statistically significant negative or positive correlations (*P < 0.05; **P < 0.01)

To elucidate the relationships among the gut microbiota, lipid metabolism, and DEGs (differentially expressed genes), heatmaps were generated through Pearson correlation analysis. In terms of the correlation between lipid metabolites and DEGs, six lipid metabolites—PE(18:1(9Z)/0:0), PE(P-18:0/22:6(4Z,7Z,10Z,13Z,16Z,19Z)), PE-NMe2(15:0/22:2(13Z,16Z)), (Z)−13-octadecenoic acid, humilixanthin, crocin 3,3-O-sulfogalactosylceramide (d18:1/20:0), and pregnanetriol—showed a significant positive correlation with changes in the expression of the chloride channel protein 2 (CLIC2) and myophilin genes. Conversely, these metabolites exhibited a negative correlation with changes in the serine/threonine-protein kinase SBK1 (SBK1), transmembrane protease serine 9-like (TMPRSS9), and anionic trypsin genes (Fig. 6A). In the analysis of the correlation between gut bacteria and DEGs, *Cyanobacteria*, *Patescibacteria*, *Synergistota*, *Deferribacterota*, *Chloroflexi*, and the ALDH (aldehyde dehydrogenase, dimeric NADP-preferring-like) gene changes showed a positive correlation. Conversely, changes in *Cyanobacteria*, *Patescibacteria*, and the PPlase5 (peptidyl-prolyl cis–trans isomerase 5) gene exhibited a negative correlation. Furthermore, *Patescibacteria*, *Deferribacterota*, and *Chloroflexi* abundances were negatively correlated with changes in the Duoxa1 (dual oxidase maturation factor 1) gene (Fig. 6B).

## Discussion

### Salinity stress-induced changes in growth and health of shrimp

The gastrointestinal tract is closely related to the digestion and absorption of nutrients in aquatic animals, serving multiple barrier functions. The functionality of intestinal tissues correlates with their structural characteristics, where modifications such as increased villi length and epithelial cell numbers enhance the ability of the animal to digest and absorb nutrients (Chen et al., 2021a). The results indicate that in the high-salinity group (NS), an increase in intestinal folds, a reduction in the intestinal lumen space, and an increase in connective tissue numbers facilitated the linkage of shrimp intestinal tissues, promoting the absorption of nutrients and the formation of various protective barriers. Conversely, as the salinity decreases, the osmotic gradient between the shrimp body and the surrounding water changes, affecting the absorption of essential nutrients. Reduced salinity leads to stress responses in shrimp, manifested as a significant reduction in intestinal folds, suggesting a decrease in the surface area available for nutrient absorption. Thinning of the muscle layer may affect intestinal motility and overall function, thereby diminishing the gut capacity for food absorption and metabolism. Physiological indicators corroborate this viewpoint, with the NS group showing significantly lower weight gain, weight gain rate, and specific growth rate than the YJ and SZ groups.

The hepatopancreas, a key organ for metabolism and digestion, displays significant morphological and functional adaptability under changing environmental salinity. These changes not only represent the organ response to stress but also reveal regulatory mechanisms at the cellular level (Xue et al. 2022). Under low-salinity conditions (NS), the stellate structure of hepatopancreatic cells almost vanishes, the lumen structure enlarges, vacuolation increases, and the tissue edges become thinner. These morphological changes are likely due to cellular volume regulatory (VR) responses necessitated by the need to maintain osmotic balance. In an environment of decreased salinity, the osmotic pressure of the extracellular fluid decreases, prompting cells to absorb water to increase their volume, thus reducing the osmotic pressure difference between the inside and outside of the cell. Furthermore, the disappearance of stellate structures and the increase in vacuolation may reflect internal restructuring of cells to enhance their tolerance to low-salinity environments (Chen et al., 2015).

In response to morphological alterations under low-salinity stress, hepatopancreatic cells exhibit a marked increase in superoxide dismutase (SOD) activity, indicative of oxidative stress mitigation strategies to counteract elevated free radical levels and protect cellular structures (Wang et al. 2015). This antioxidative defense enhancement is a crucial cellular adaptation that preserves cell integrity and functionality. Concurrently, the upregulation of acid phosphatase (ACP) and alkaline phosphatase (AKP) activity signals metabolic adjustments essential for managing stress-induced energy demands and cellular repair (Stocker and Keaney 2004). These enzymatic changes, reflecting shifts in phosphate transport, metabolism, and membrane stability, are adaptive measures to maintain cellular homeostasis and membrane permeability in the face of environmental salinity fluctuations. Such regulation underscores the cell ability to modulate enzyme activities to bolster membrane integrity and optimize intracellular metabolite use, ensuring survival and function under challenging conditions.

### Response of the intestinal microbiota to high and low salinity

#### Microbial composition under different salinity conditions

Salinity is a critical ecological factor that influences the physiological and metabolic functions of *Penaeus monodon*, with significant implications for their growth, reproduction, and distribution. This study explores the changes in the intestinal microbiota of *P. monodon* across a gradient of salinity conditions, highlighting the dynamic shifts in microbial communities that contribute to the shrimp ability to adapt to varying environmental stresses.

#### High salinity conditions

Under high-salinity environments, there is a pronounced increase in the abundance of the phyla Proteobacteria and Cyanobacteria. These groups are pivotal in adapting to saline stress through several mechanisms:

- Proteobacteria enhance the shrimp resilience by engaging in nutrient cycling, pathogen defense, and stress response (Herrou et al. 2012). Specific species within this phylum are noted for their role in modulating immune responses, contributing to the nitrogen and carbon cycles, and producing bioactive substances that mitigate physiological damages caused by salinity stress.
- Cyanobacteria contribute significantly by using their photosynthetic capabilities to produce oxygen and organic compounds, which support the survival and enhance the redox balance of the microbial community (Rezayian et al. 2019). Notable among them is *Cyanobium PCC-6307*, which plays a critical role in maintaining osmotic balance and boosting immune system efficacy under stress (Karandashova and Elanskaya 2005).

The Rhodobacteraceae family, including genera like *Parasphingopyxis* and *Rhodobacter azotoformans*, also shows strong adaptability in high salinity, maintaining biomass and providing significant microbial support that aids the shrimp survival (Egamberdieva, 2018).

#### Low salinity conditions

In contrast, low-salinity environments are dominated by Firmicutes, Bacteroidota, and Actinobacteriota, indicating a distinct shift in the microbial landscape that influences various physiological aspects of the shrimp:

- Bacteroides play a crucial role in degrading complex polysaccharides, providing essential nutrients and helping maintain the balance of the intestinal immune system, critical for the shrimp health in less saline waters (Ikeyama et al., 2020).
- Collinsella species are involved in producing deoxycholic acid, which aids liver function and bile acid excretion, essential for maintaining intestinal and overall health (Narushima et al., 2006).
- Phascolarctobacterium, by participating in sugar breakdown and succinate metabolism, significantly contributes to the energy supply and metabolic balance, influencing the hos metabolic regulation and glucose homeostasis (Fernández-Veledo and Vendrell 2019).

#### Gut microbiome analysis and its correlations with physiological and biochemical phenotypes

Gut microbiome analysis has shed light on how *P. monodon* leverages its intestinal microbiota to adapt to different saline environments. Under high-salinity conditions, significant alterations in the structure of the gut microbial community occur, particularly with increased relative abundances of *Proteobacteria* and *Cyanobacteria*. These microbial enhancements are closely associated with improved osmoregulatory capabilities and immune functions of the shrimp, subsequently affecting their physiological and biochemical phenotypes, such as enhanced growth rates and increased body weight (De Vadder et al. 2016).

Specifically, certain members of Proteobacteria may engage in nitrogen cycling and organic matter decomposition, providing essential nutrients to shrimp, while the photosynthetic byproducts of *Cyanobacteria* could serve as additional energy sources. The functions of these microbes include bolstering energy acquisition and utilization by shrimp under high salinity, aiding in maintaining their normal physiological state and growth development. Moreover, these two major microbial classes might also enhance shrimp disease resistance by producing antibiotics and other bioactive substances, indirectly influencing shrimp growth and survival rates. In low-salinity environments, changes in the abundances of *Firmicutes* and *Bacteroidetes* may reflect the role of the gut microbiota in regulating energy usage and lipid metabolism. These changes correlate with adjustments in physiological and biochemical phenotypes, such as changes in weight gain rates and specific growth rates. By modulating the composition of the gut microbiome, *P. monodon* may optimize the digestion and absorption of food, especially in lower-salinity environments, by maintaining efficient energy intake and nutrient absorption (Ikeyama et al., 2020).

The correlation between the gut microbiome composition and physiological and biochemical phenotypes elucidates how *P. monodon* adapts to changes in environmental salinity by modulating the diversity and abundance of the intestinal microbiota. This adaptive mechanism encompasses not only the optimization of energy acquisition and utilization but also the enhancement of immune functions and disease resistance, thereby augmenting shrimp survival. Consequently, the composition and functionality of the gut microbiota are pivotal to the overall health and adaptability of *P. monodon*. For the aquaculture industry, modulating the salinity of the cultivation environment to optimize the composition of the gut microbiome represents an effective strategy for enhancing cultivation efficiency and the health of shrimp species.

### Hepatopancreatic transcriptomic response to salinity stress

#### Hepatopancreatic transcriptome

This section of the discussion focuses on the transcriptomic response of the hepatopancreas in *P. monodon* to salinity stress, as revealed through RNA-Seq technology. The intricate analysis of metabolic reactions in the hepatopancreas under varying salinity conditions, particularly via KEGG pathway analysis, underscores the complexity and subtlety of the effects of salinity stress on the metabolic pathways of *P.* monodon. This study highlights how high salinity predominantly affects pathways such as cysteine and methionine metabolism, complement and coagulation cascades, glycolysis/gluconeogenesis, and tryptophan metabolism, reflecting cellular adaptation to environmental changes through metabolic regulation.

Cysteine and methionine, which are sulfur-containing amino acids, are not only fundamental components for protein synthesis but also precursors for antioxidants such as glutathione. The activation of these metabolic pathways under high salinity conditions illustrates the cellular response to increased oxidative stress, enhancing antioxidative defense to maintain redox balance (Kim et al. 2014). Simultaneously, alterations in glycolysis/gluconeogenesis pathways suggest that cells might adjust their energy metabolism to cope with high salinity stress, ensuring the maintenance of ATP levels required for cellular activities under energy-limited conditions (Saito 2000).

The study of salinity stress in *P. monodon* through the analysis of differentially expressed genes (DEGs) has explored how salinity variations affect lipid metabolism pathways by regulating the expression of specific genes, thereby revealing the molecular mechanisms underlying *P. monodon* adaptation to salinity stress environments. This regulatory mechanism includes fine-tuning lipid synthesis, degradation, and transport processes, reflecting complex adaptive strategies to environmental stress.

Enhanced lipid synthesis as an energy reserve strategy, adjustments in cell membrane composition and properties, the regulation of antioxidative defense and inflammatory responses, and the impact on membrane phospholipid composition and apoptotic processes all indicate how *P. monodon* adapts its expression of lipid metabolism-related genes to cope with high and low salinity conditions (Duan et al., 2015; Ye et al., 2009). This includes increasing energy reserves, adjusting the physicochemical properties of cell membranes, coping with oxidative stress and inflammatory responses, and affecting the apoptotic process.

#### Hepatopancreatic metabolism and its correlations with physiological and biochemical phenotypes

Metabolomic analysis of the hepatopancreas revealed that the physiological adaptability of *P. monodon* under different salinity conditions is closely related to adjustments in its metabolic pathways. Metabolomic data revealed significant metabolic changes in the hepatopancreas under high-salinity conditions, especially in lipid metabolism, amino acid metabolism, and antioxidative defense. For instance, notable changes in lipid metabolic products such as 3-O-sulfogalactosylceramide, phosphoethanolamine, and CDP-diacylglycerol assist in maintaining the stability and permeability of cellular membranes, thus preserving normal cellular functions under salinity stress (Farwanah and Kolter 2012). Additionally, increased activity of the SOD enzyme reflects an increase in the antioxidative defense mechanism, a critical adaptive response of shrimp to oxidative damage in low-salinity environments (St Germain et al. 2022).

Taken together, these findings reveal the complexity of *P. monodon* molecular adjustments to salinity stress, underscoring the integral roles of lipid metabolism, antioxidative defense, and energy balance in facilitating shrimp adaptation to its changing environment.

### Hepatopancreatic metabolomic response to salinity stress

#### Hepatopancreatic metabolomics

The hepatopancreatic metabolomics analysis further confirmed that salinity stress significantly affects the metabolic functions of *P. monodon*, especially in terms of amino acid metabolism. Under high salinity stress, the pathways most affected included bile secretion, D-arginine and D-ornithine metabolism, choline metabolism and arginine and proline metabolism. Under low-salinity stress, the impacted pathways primarily involved bile secretion, D-arginine and D-ornithine metabolism, choline metabolism and glutathione metabolism.

Under high salinity stress conditions, the pathway of bile secretion in *P. monodon* is significantly affected (Schonewille et al. 2016). This suggests that salinity stress may disrupt the synthesis and secretion of bile acids, which are crucial for the digestion and absorption of lipids. Bile acids emulsify fats, increasing the accessibility of fatty acids for further breakdown and absorption (Rivera-Perez and García‐Carreño, 2011). Salinity stress can affect the normal synthesis and regulation of bile acids, thereby impacting lipid metabolism and reducing the effective acquisition of energy.

Furthermore, changes in D-arginine and D-ornithine metabolism and arginine and proline metabolism pathways under salinity stress could affect energy metabolism and the nitrogen cycle. Arginine, a key metabolic node involved in the synthesis of nitric oxide, and proline, which play significant roles in the cellular response to environmental stress, particularly in osmotic regulation and antioxidative defense, indicate that these metabolic pathway changes might reflect adjustments in energy production and use, as well as the maintenance of internal environmental stability in *P. monodon* (Rus-Alvarez and Guerrier, 1994).

Additionally, alterations in choline metabolism could reflect alterations in cell membrane composition and signaling mechanisms (Glunde and Serkova 2006). Choline, an essential component of phospholipids, is vital for maintaining the structure and function of cell membranes (Gorin et al. 2012). Under environmental stress, cells might adjust their membrane phospholipid composition to change their fluidity and permeability, thereby protecting the cell from damage and regulating signaling pathways to adapt to external changes.

Under low-salinity stress conditions, in addition to impacts similar to those under high-salinity stress, changes in the glutathione metabolism pathway are noteworthy. This pathway adjustment highlights the activation of antioxidative defense mechanisms. Glutathione, a key antioxidant, plays a crucial role in defending against oxidative stress and protecting cells from free radical damage (Cnubben et al. 2001). Low-salinity environments may increase intracellular oxidative stress, promoting the synthesis and utilization of glutathione (Chakravarthi et al., 2006) and thereby activating cellular antioxidative responses to maintain redox balance and cell integrity.

The identification of differentially metabolized compounds (DMs) underscores how *P. monodon* modulates membrane lipid metabolism, osmoregulatory defenses, and antioxidative responses to salinity shifts. This study delineates adaptation mechanisms, highlighting significant DM alterations in both high- and low-salinity stress environments (Lopalco et al. 2013). Membrane lipid adjustments, marked by the upregulation of compounds such as 3-O-sulfogalactosylceramide and PE-NMe2, enhance membrane stability and permeability, which are critical for maintaining osmotic balance amidst salinity changes (Ikbal et al., 2014). Additionally, the increase in the levels of specific sphingolipids and phosphatidylethanolamines underlines shrimp strategy to preserve physiological functions by fine-tuning membrane lipid composition. The increase in the levels of antioxidative compounds such as CDP-DG, *soyasaponin V*, and *notoginsenoside J* indicates robust activation of antioxidative defenses, mitigating oxidative stress and potentially modulating intracellular inflammation and signaling pathways, thereby bolstering shrimp adaptability and survival under varying salinity levels (Sharma and Sharma 2018).

#### Transcriptomic analysis and its correlation with physiological and biochemical phenotypes

Transcriptomic data provide further molecular-level insights into the adaptability mechanisms of *P. monodon* under different salinity conditions. The analysis revealed the upregulation of genes related to lipid metabolism, such as the ATP-dependent RNA helicases DDX27, SC5DL, and ECH1, under high salinity, highlighting the importance of lipid metabolism in the response to high-salinity stress (Busswinkel et al. 2018). Additionally, significant changes in the expression of genes related to amino acid metabolism and energy balance suggested that *P. monodon* adapts to changes in salinity by adjusting pathways involved in amino acid metabolism and energy production. Specifically, KEGG pathway analysis highlighted the adjustment of key metabolic pathways, such as fatty acid metabolism, methylenetetrahydrofolate reduction, and arginine and proline metabolism pathways. These metabolic pathway adjustments directly affect the growth rate, weight gain, and specific growth rate of shrimp, demonstrating the correspondence between molecular-level regulation and physiological phenotypes (Chen et al. 2015).

The integration of specific hepatopancreatic metabolomics and transcriptomics data revealed how *P. monodon* finely tunes key genes and metabolites involved in lipid and amino acid metabolism to adapt to environmental changes induced by salinity stress. Such adjustments in metabolic pathways not only preserve cellular energy and redox balance but also enhance shrimp tolerance to salinity fluctuations, thereby promoting growth and survival. This detailed exploration underscores the critical role of metabolic pathway modulation in enabling *P. monodon* to navigate the challenges presented by varying salinity levels, highlighting the intricacies of its adaptive strategies at the molecular level.

### Integrated multi-omics and phenotypic analysis

By integrating findings from gut microbiome analyses, hepatopancreatic metabolomics, transcriptomics, and physiological and biochemical phenotype analyses, this study elucidates a complex adaptive mechanism by which *P. monodon* navigates salinity stress. This adaptation is mediated through a dynamic interplay between microbiota composition changes, lipid metabolism regulation, and differential gene expression, which collectively fine-tune the physiological state of shrimp to varying salinity conditions.

Pearson correlation analysis between lipid metabolites and DEGs revealed significant interactions where specific lipid metabolites were positively correlated with genes integral to ion balance and muscle function, such as CLIC2 and myophilin, under salinity stress. This suggests that lipid metabolism adjustments directly influence the expression of genes crucial for maintaining cellular homeostasis and physiological functions in *P. monodon* (Jiang et al., 2019). Conversely, a negative correlation with genes involved in protein processing and stress responses, including SBK1, TMPRSS9, and anionic trypsin, underscores the intricate relationship between lipid metabolism and cellular stress mechanisms (Okumura et al. 2006).

Furthermore, this study highlights how changes in microbial communities, particularly those of *Cyanobacteria*, *Patescibacteria*, *Synergistota*, *Deferribacterota*, and *Chloroflexi*, are linked to the regulation of genes associated with antioxidative defense, energy metabolism, and osmoregulation. This finding points to a pivotal role of the gut microbiome in modulating *P. monodon* adaptative responses to salinity through direct impacts on metabolic processes and gene expression (Hieu et al. 2022).

In high-salinity environments, the increase in *Proteobacteria* and *Cyanobacteria* supports enhanced osmoregulation and immune functions and contributes to nutrient provision and energy acquisition, facilitating improved growth rates and increased body weight in *P. Monodon* (Duan et al. 2020). These microbial shifts reflect a strategic adjustment to optimize energy utilization and lipid metabolism in response to environmental stress, further evidenced by significant metabolic changes in the hepatopancreas. Notably, alterations in lipid metabolites such as 3-O-sulfogalactosylceramide and phosphoethanolamine help maintain cellular membrane stability and function, while upregulated antioxidative enzymes such as SOD are a critical adaptation to oxidative stress at varying salinity levels (Ikbal et al., 2014).

The transcriptomic data enrich the understanding of this phenomenon, showing the upregulation of genes related to lipid metabolism and adjustments in amino acid metabolism and energy production pathways, aligning with the metabolic demands imposed by salinity stress. This gene expression modulation directly influences *P. monodon* growth performance and survival strategies, demonstrating a tight correlation between metabolic and transcriptomic adaptations and the physiological phenotype of the shrimp.

### Adaptive regulation of lipid metabolism in *P. monodon* under salinity stress

In the face of reduced salinity, *P. monodon* initiates a sophisticated adaptation response, underpinned by an orchestrated modulation of transcriptional activity, metabolic pathways, and microbiome composition. The hepatopancreatic transcriptome undergoes significant remodeling, where genes like ECH1, pivotal in the β-oxidation pathway, exhibit increased expression, thereby boosting the conversion of LC-acyl-CoA to Enoyl-CoA and ensuring a continued supply of acetyl-CoA for energy production under osmotic stress (Busswinkel et al. 2018). Concurrently, the upregulation of genes such as SC5DL and DD27, which are integral to cholesterol biosynthesis and lipid metabolism respectively, suggests a reinforcement of membrane structure and fluidity through the accrual of cholesterol and complex lipids (Chen et al. 2015).

**Fig. 7:**
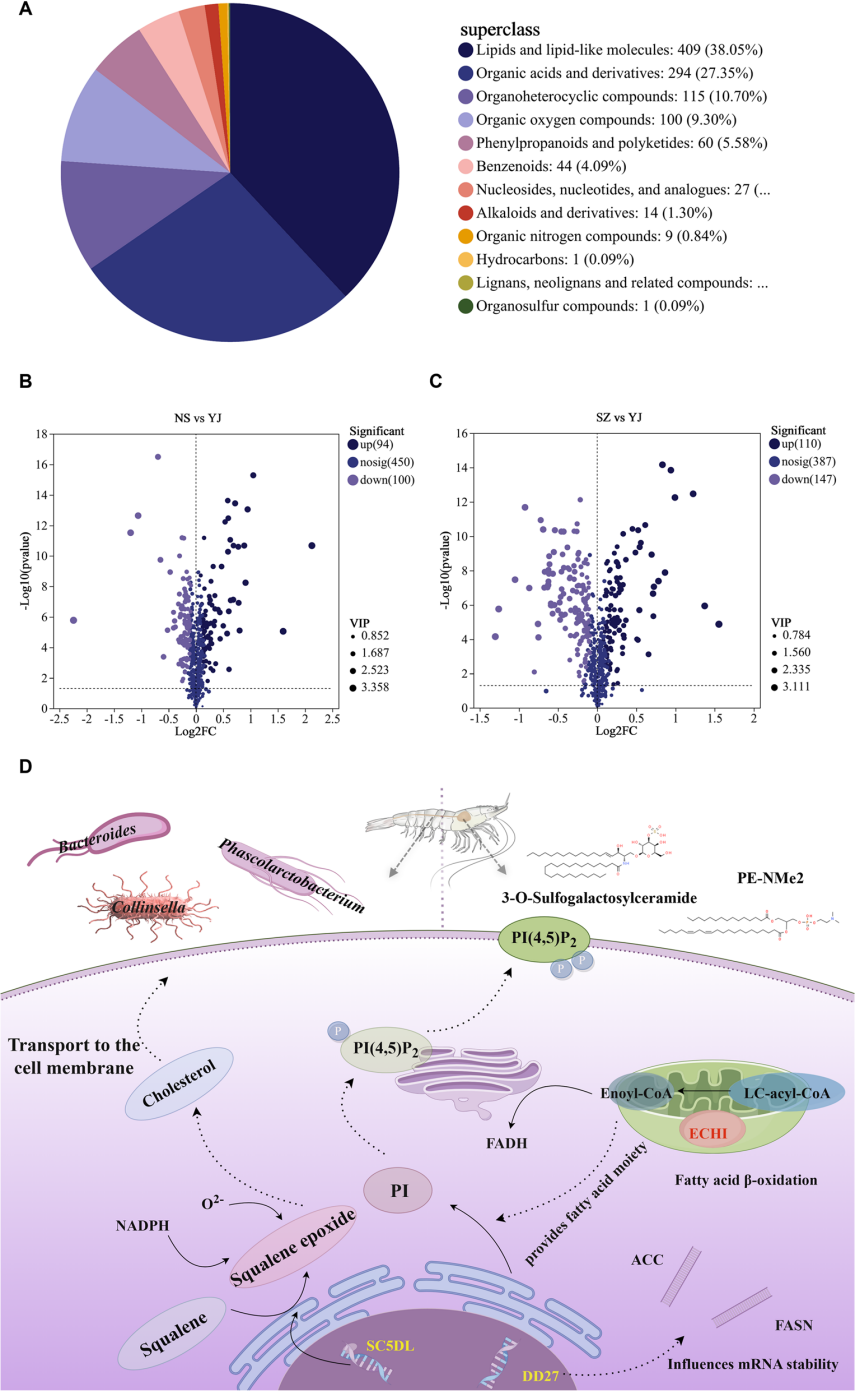
Metabolomic Analysis of Hepatopancreatic Responses to Salinity Stress in *P. monodon* and Mechanistic Insights into Low Salinity Adaptation: **A** Classification of compounds according to the Human Metabolome Database (HMDB); **B**-**C** Volcano plots comparing differentially abundant metabolites in the hepatopancreas of* P. monodon* between the two groups under different salinity stresses; **D** Multi-omics Hypothesis on the Mechanism of Salinity Tolerance in *P. monodon* via Lipid Signaling Pathways

Furthermore, alterations in the transcriptome extend to genes like FASN and ACC, which are implicated in fatty acid synthesis, reflecting a dynamic adjustment to maintain cellular homeostasis. These transcriptional adjustments are mirrored in the metabolome, where an increased abundance of specific lipids such as phosphatidylinositols, signified by PI(4,5)P_2_ in the diagram, support membrane integrity and signal transduction, essential for cell survival under salinity flux (Guo et al., 2019). The lipidome reconfiguration also encompasses the elevation of 3-O-Sulfogalactosylceramide, a sphingolipid that contributes to the stabilization of cell membranes against osmotic imbalance. Based on multi-omics data analysis, we have constructed a hypothetical model of the salinity tolerance mechanism in *P. monodon* through lipid signaling pathways (Fig. 7).

## Conclusion

This comprehensive study elucidates the intricate mechanisms of *P. monodon* adaptation to salinity fluctuations by integrating gut microbiome analysis, hepatopancreatic transcriptomics, and metabolomics with phenotypic observations. Shrimp employ a multifaceted molecular strategy to negotiate environmental stress, highlighted by a significant restructuring of the gut microbiome under high salinity, with a notable increase in *Proteobacteria* and *Cyanobacteria*, which bolster osmoregulatory and immune functions. This microbial shift is aligned with substantial adjustments in lipid metabolism within the hepatopancreas, as revealed by transcriptomic and metabolomic analyses, to preserve cellular integrity and maintain energy balance under osmotic stress. This study also revealed that low salinity triggers extensive responses across metabolic and transcriptomic profiles, affecting pathways related to oxidative stress, immune function, and cellular repair mechanisms. Such adaptations are crucial for maintaining homeostasis and underscore the dynamic interaction between microbiome diversity and shrimp metabolic strategies. Furthermore, phenotypic data, including growth rates and specific growth rate variations across different salinity levels, provide a tangible link between the observed molecular adaptations and *P. monodon* ability to thrive at varying salinities. By integrating these multiomics data with phenotypic trait data, this research provides a holistic picture of the genetic, metabolic, and microbial underpinnings of salinity adaptation in *P. monodon*, offering invaluable insights for enhancing aquaculture practices and resilience to environmental challenges.

## Materials and methods

### Experimental setup and sampling methodology

#### Experimental setup

Juvenile *Penaeus monodon* specimens were sourced from the Shenzhen Experimental Base of the South China Sea Fisheries Research Institute, Chinese Academy of Fishery Sciences. All specimens originated from a uniform genetic stock to eliminate genetic variability as a confounding factor.

The experimental setup comprised nine artificial concrete ponds divided among three locations in Shenzhen (SZ), Nansha (NS), and Yangjiang (YJ), chosen for their differing natural salinity levels. These ponds were configured as follows:

- Three ponds at each location with dimensions of 5 × 4.2 × 1.4 m in SZ, 4.2 × 4.8 × 1.4 m in NS, and 5 × 5 × 1.4 m in YJ.
- Each pond was stocked with 300 juvenile shrimp, maintaining consistent density across varying conditions.

Salinity levels were adjusted and maintained using locally sourced, purified seawater at 30 ppt for SZ, 20 ppt for YJ, and 5 ppt for NS. Identical systems for water circulation, aeration, and temperature control were installed in each pond to standardize environmental conditions apart from salinity. The shrimp were fed a standardized diet formulated for optimal growth under different salinity conditions, distributed equally across all locations (Figure S2).

#### Sampling methodology

Cultivation began on May 12, 2023, and continued for 62 days, concluding on July 12, 2023. Samples were collected for analysis from three different salinity conditions: 5 ppt, 20 ppt, and 30 ppt. Nine artificial concrete ponds were established, with three ponds allocated to each salinity treatment. The initial average body length of the shrimp was 5.43 ± 0.14 cm, and the average initial weight was 0.962 ± 0.072 g. Water quality parameters, including temperature, pH, and dissolved oxygen levels, were measured throughout the cultivation period (Table S1). From each pond, a total of 30 individual shrimps were randomly selected, resulting in 90 shrimps per salinity condition (30 shrimps from each of the three ponds). This method provided a robust sample size, ensuring comprehensive representation of each treatment group. The selected shrimps were pooled from each pond to create three biological replicates per salinity treatment, culminating in a total of nine biological replicates for transcriptomic and metabolomic analyses across the three salinity levels (Figure S1).

#### Replication and data collection

For more detailed analysis, an additional set of three pools (totaling nine pools) was sampled from each location, providing a total of eighteen samples for hepatopancreatic tissue analysis.

Environmental parameters, including water temperature, pH, and salinity, were monitored bi-weekly. Concurrently with the biological samples, water and sediment samples were collected with four replicates per medium at each site to assess environmental influences on shrimp health and microbial composition.

Environmental water samples were filtered through a 0.2 µm membrane filter to remove particulate matter. The filters were then placed in cryogenic vials and immediately frozen at −80 °C for preservation and transport. Soil samples were collected and stored at −80 °C to maintain their integrity before analysis. For biological samples, including the intestine, hepatopancreas, and muscle tissues of *Penaeus monodon*, sampling was conducted on ice to minimize degradation. Each tissue sample was weighed and then homogenized in phosphate-buffered saline (PBS) at a ratio of 1:10 (w/v) using a motorized tissue homogenizer to create a uniform suspension. The homogenates were subsequently aliquoted and stored at −80 °C until further analysis.

### Measurement of shrimp growth indicators

Feeding ceased 24 h prior to sampling. Sixteen *P. monodon* specimens from each parallel group were randomly selected for counting and weighing to calculate various growth indicators. The formulas for calculating growth indicators are as follows:

- Weight gain (WG) = W_f_-W_i_;
- Weight gain rate (WGR) = 100 (W_f_-W_i_)/W_i_;
- The SGR was calculated as (lnW_f_-lnW_i_)/t × 100%, where t represents the number of days of the experiment, W_f_ is the total mass of the shrimp at the end of the experiment, and W_i_ is the total mass of the shrimp at the beginning of the experiment.

### Intestinal and hepatopancreatic tissue sectioning

Specimens of intestinal and hepatopancreatic tissues were fixed in 4% paraformaldehyde for 24 h, followed by washing, dehydration, clarification, and paraffin embedding. Continuous 5 μm thick paraffin sections were then prepared and stained with hematoxylin and eosin (H&E) for microscopic observation.

### Determination of biochemical indicators

Tissues from the gills, hepatopancreas, and intestine were homogenized in physiological saline at a specific weight/volume ratio under ice-water bath conditions. Following centrifugation, the supernatant was collected and analyzed for superoxide dismutase (SOD), catalase (CAT), acid phosphatase (ACP), alkaline phosphatase (AKP), and 1-aminocyclopropane-1-carboxylate oxidase (ACO) activities according to the protocols described in the kits provided by Nanjing Jiancheng Bioengineering Institute.

### Gut microbiome analysis

Total microbial genomic DNA from intestinal samples was extracted using the TAB/SDS method. Sequencing primers, including barcodes, were synthesized to target specific regions. Preliminary quantification of PCR products was performed via electrophoresis, followed by precise quantification using the QuantiFluor™-ST system (https://www.promega.com/). PCR products were then pooled in ratios aligned with sequencing requirements. Illumina adapter sequences were applied to the target DNA regions via PCR (Edgar 2013). Sequencing was performed on the Illumina platform, with detailed protocols available at Illumina official documentation (Edgar et al. 2011). Sequence optimization and redundancy reduction were conducted to minimize the computational load during analysis, as detailed in Drive5 (https://drive5.com/usearch/manual/dereplication.html) (Schloss et al. 2011). Sequences with ≥ 97% similarity were clustered into OTUs, and chimeras were eliminated to identify representative sequences (Segata et al. 2011). Mapping of sequences to OTU representatives facilitated the generation of OTU tables for taxonomic classification at various levels using the RDP classifier Bayesian algorithm (RDP Classifier 2.11 https://sourceforge.net/projects/rdp-classifier/), with a confidence threshold of 0.7. Comprehensive statistical analyses were performed using R software packages (Duan et al. 2021; Kim et al. 2015).

### Alpha diversity analysis

Alpha diversity indices offer insights into the community richness, evenness, diversity, and coverage. Richness indices include observed richness (Sobs), Chao1 estimator, ACE estimator, Jackknife estimator, and bootstrap estimator. Evenness is captured by the Pielou evenness index, Simpson evenness measure, Shannon evenness measure, Heip evenness metric, and Smith and Wilson evenness metric. Diversity is reflected in the Shannon diversity index, Simpson diversity index, non-parametric Shannon diversity index, Berger-Parker dominance index, inverse Simpson index, and Q statistic. The Good coverage index reflects community coverage.

For the analysis, operational taxonomic units (OTUs) are clustered at a 97% similarity level, and diversity indices are calculated using mothur software (version v.1.30.2). Inter-index variation is tested via Student t-test, Welch t-test, and Wilcoxon rank-sum test, employing R language (version 3.3.1) with the boot (1.3.18) and stats (3.3.1) packages.

### Intestinal transcriptome analysis

Total RNA was isolated from tissue samples, and its concentration and purity were assessed via a Nanodrop2000. RNA integrity was verified through agarose gel electrophoresis and an Agilent 2100 bioanalyzer, and the RNA integrity number (RIN) was determined. The construction of sequencing libraries utilized 1 μg of high-quality RNA samples, employing the TruSeq™ RNA Sample Preparation Kit from Illumina (Illumina TruSeq). Following quantification with TBS380, sequencing was conducted on the Illumina HiSeq platform, adopting a PE library with a read length of 2 × 150 bp. Reference genome-based assembly and annotation of newly identified transcripts were performed using Cufflinks (http://cole-trapnelllab.github.io/cufflinks/) or StringTie (http://ccb.jhu.edu/software/stringtie/), integrating sequence function information to elucidate regulatory mechanisms.

### KEGG pathway enrichment analysis

Similarly, KEGG PATHWAY enrichment was carried out using custom R scripts that incorporate statistical rigor analogous to that used in the GO enrichment analysis. KEGG pathways, which map genes to higher order functional hierarchies, provide insight into how sets of genes or transcripts are involved in specific metabolic pathways or molecular interaction networks. Pathways with an adjusted *P*-value (Padjust) less than 0.05, post-correction for multiple testing, were deemed to have significant enrichment.

### Hepatopancreatic metabolomic analysis

Metabolites were extracted from hepatopancreatic samples using a methanol: chloroform solvent system, followed by homogenization, centrifugation, and supernatant collection. Metabolomic profiling was conducted using Liquid Chromatography-Mass Spectrometry (LC–MS/MS). Data preprocessing included alignment, normalization, and peak identification. Principal Component Analysis (PCA), Partial Least Squares Discriminant Analysis (PLS-DA), and Orthogonal Partial Least Squares Discriminant Analysis (OPLS-DA) were used to identify differentially abundant metabolites, focusing on those with high Variable Importance in Projection (VIP) scores. Volcano plots visualized the significance and magnitude of metabolite changes. Metabolic pathways affected by experimental conditions were elucidated using the KEGG PATHWAY and iPath3.0 databases.

### VIP score calculation

In metabolomic studies, the integration of univariate (parametric and non-parametric tests) and multivariate (PCA and PLS-DA) statistical analyses is pivotal for identifying differential metabolites between groups. The study employs PCA and PLS-DA to assess global variances, followed by OPLS-DA (or PLS-DA for overfitting scenarios) combined with univariate metrics (Fold change and p-values) for metabolite selection. Differential metabolites are visualized using volcano plots. The model predictive accuracy is ensured through sevenfold cross-validation in OPLS-DA/PLS-DA, with VIP scores pinpointing key metabolites for classification, thereby identifying potential biomarkers. This streamlined approach effectively deciphers metabolic distinctions within complex datasets.

### Multi-omics integrated analysis

Differential biomarkers and corresponding expression levels from the transcriptome, metabolome, and intestinal microbiome were subjected to correlation analysis using the Pearson correlation coefficient algorithm. The analysis was conducted using GenesCloud (www.genescloud.cn).

### Data processing and statistical analysis

The experimental data were statistically analyzed using GraphPad software. Comparisons among groups were made using one-way analysis of variance (ANOVA) followed by Duncan multiple range test. All the data are presented as the mFFean ± standard deviation (SD). A *P* value less than 0.05 was considered to indicate statistical significance.

## Supplementary Information

Supplementary Material 1: Figure S1: Sampling and Data Collection Flowchart for Penaeus monodon Study Across Three Locations. Figure S2: Images of shrimp farming ponds at three different locations.

Supplementary Material 2: Table S1. Water Quality Parameters for P. monodon.

## Data Availability

Not applicable.

## Abbreviations

ACP: Acid phosphatase
ACO: 1-Aminocyclopropane-1-carboxylate oxidase
AKP: Alkaline phosphatase
CAT: Catalase
CLIC2: Chloride Intracellular Channel 2
DDX27: DEAD-box helicase 27
DEGs: Differentially expressed genes
DMs: Differentially metabolized compounds
ECH1: Enoyl Coenzyme A Hydratase 1
FASN: Fatty Acid Synthase
GO: Gene Ontology
KEGG: Kyoto Encyclopedia of Genes and Genomes
NS: Nansha
*P. monodon*: *Penaeus* *monodon*
SBK1: SH3-binding domain kinase 1
SC5DL: Sterol-C5-desaturase
SOD: Superoxide dismutase
SZ: Shenzhen
TMPRSS9: Transmembrane Serine Protease 9
YJ: Yangjiang
