# Supplementary figures and images for: Omics analysis of Penaeus monodon in response to salinity changes
Source: Stress Biol. 2025 Feb 27;5(1):17. doi: 10.1007/s44154-024-00207-4 (PMC11865391; doi:10.1007/s44154-024-00207-4)

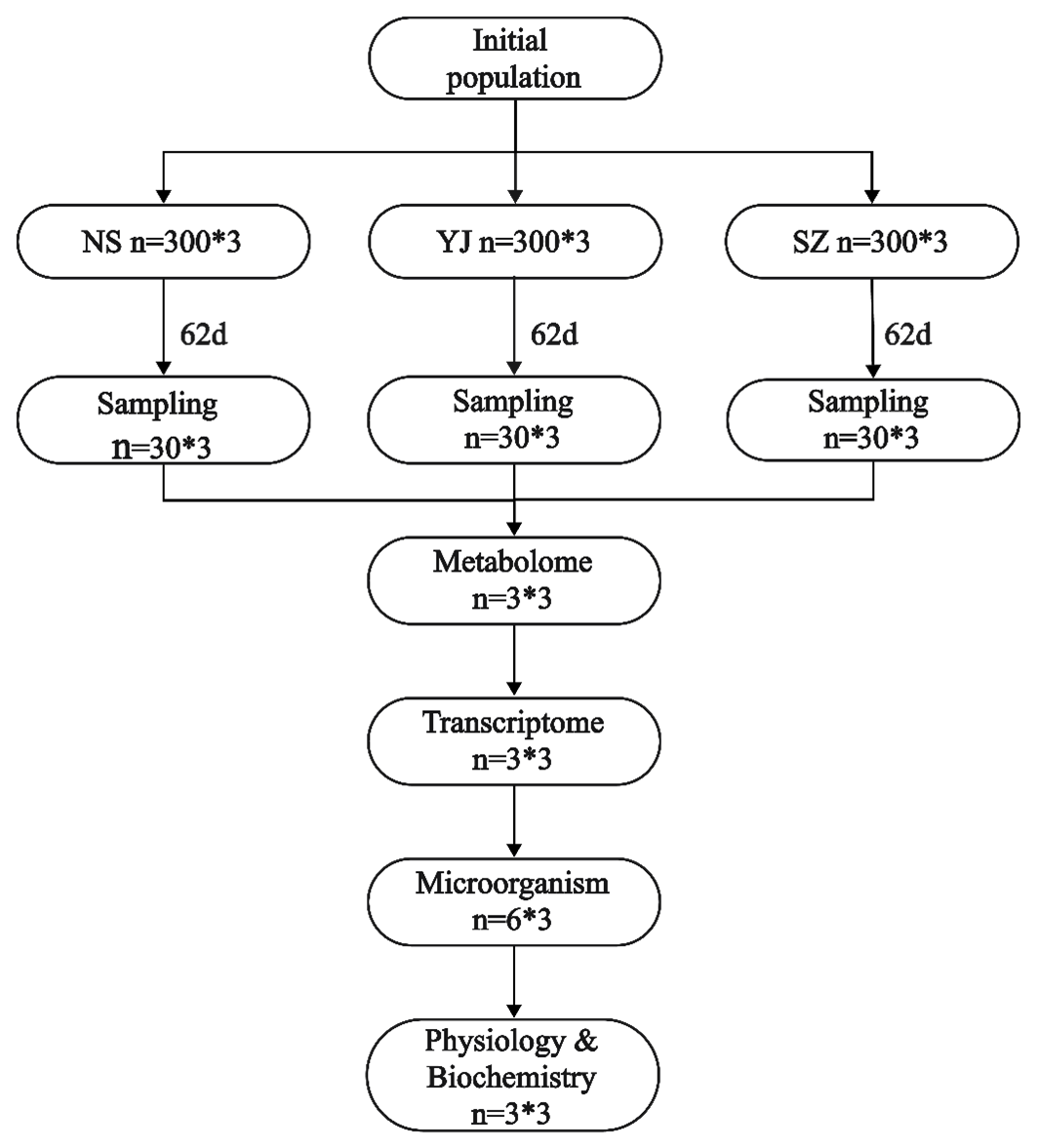

Supplement: Supplementary file 1 — Supplementary Material 1: Figure S1: Sampling and Data Collection Flowchart for Penaeus monodon Study Across Three Locations. Figure S2: Images of shrimp farming ponds at three different locations. [file 44154_2024_207_MOESM1_ESM.zip › 44154_2024_207_MOESM1_ESM/Fig.S1_ESM.png]

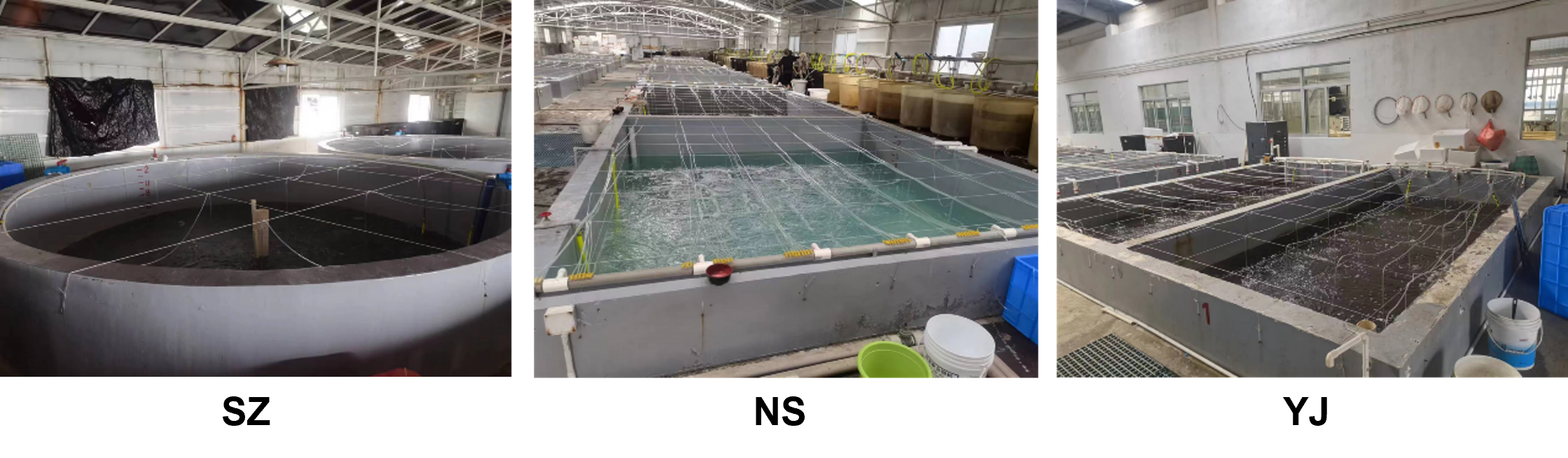

Supplement: Supplementary file 1 — Supplementary Material 1: Figure S1: Sampling and Data Collection Flowchart for Penaeus monodon Study Across Three Locations. Figure S2: Images of shrimp farming ponds at three different locations. [file 44154_2024_207_MOESM1_ESM.zip › 44154_2024_207_MOESM1_ESM/Fig.S2_ESM.png]
