# Supplementary material for: Omics analysis of Penaeus monodon in response to salinity changes
Source: Stress Biol. 2025 Feb 27;5(1):17. doi: 10.1007/s44154-024-00207-4 (PMC11865391; doi:10.1007/s44154-024-00207-4)
Supplement: Supplementary file 2 — Supplementary Material 2: Table S1. Water Quality Parameters for P. monodon. [file 44154_2024_207_MOESM2_ESM.docx]

**Table S1.** Water Quality Parameters for *P. monodon*

| Measurement Date | Location | Temperature (°C) | | Dissolved Oxygen (mg/L) | pH |
| --- | --- | --- | --- | --- | --- |
| May 12, 2023 | SZ | | 28.3 | 6.4 | 7.9 |
|  | YJ | | 28.9 | 6.8 | 7.9 |
|  | NS | | 29.1 | 6.2 | 8.1 |
| May 26, 2023 | SZ | | 29.2 | 6.3 | 7.9 |
|  | YJ | | 28.8 | 6.5 | 7.9 |
|  | NS | | 30.1 | 6.7 | 8.2 |
| June 9, 2023 | SZ | | 29.4 | 6.9 | 8.1 |
|  | YJ | | 28.9 | 6.6 | 7.8 |
|  | NS | | 29.5 | 6.9 | 8.0 |
| June 23, 2023 | SZ | | 29.6 | 6.3 | 8.0 |
|  | YJ | | 29.2 | 6.7 | 7.9 |
|  | NS | | 30.4 | 6.6 | 8.0 |
| July 7, 2023 | SZ | | 29.7 | 6.5 | 8.1 |
|  | YJ | | 29.5 | 6.8 | 8.0 |
|  | NS | | 29.9 | 6.3 | 8.0 |
| July 12, 2023 | SZ | | 29.8 | 6.2 | 7.9 |
|  | YJ | | 29.6 | 6.7 | 8.0 |
|  | NS | | 29.9 | 6.4 | 7.9 |
